# Major combined electrolyte deficiency during therapy with low-dose Cisplatin, 5-Fluorouracil and Interferon alpha: report on several cases and review of the literature [ISRCTN62866759]

**DOI:** 10.1186/1471-2407-6-128

**Published:** 2006-05-10

**Authors:** Katrin Hoffmann, Angela Marten, Katja Lindel, Stefan Fritz, Dirk Jager, Markus W Buchler, Jan Schmidt

**Affiliations:** 1 Department of Surgery, University of Heidelberg, Im Neuenheimer Feld 110, 69120 Heidelberg, Germany; 2 National Centre of Tumor Diseases, University of Heidelberg, Im Neuenheimer Feld 350, 69120 Heidelberg, Germany

## Abstract

**Background:**

Low-dose Cisplatin and Interferon alpha treatment of solid tumors rarely has been associated with severe hypocalcaemia. To the authors knowledge the phenomenon has not been reported previously in patients with pancreatic carcinoma.

**Case presentation:**

A patient with resected adenocarcinoma of the pancreas was treated with adjuvant radio-chemo-immunotherapy using a combination of low-dose Cisplatin, 5-Fluorouracil and Interferon alpha together with external beam radiation. Severe hypocalcaemia without signs of acute renal failure or electrolyte disturbance occurred within 2 days at the 4th week of treatment and required intensive care treatment.

**Conclusion:**

Combination of biological and cytotoxic therapies may increase the incidence of severe hypocalcaemia in pancreatic cancer. Oncologists should remain attentive of this problem as more highly active regimes become available.

## Background

Hypocalcaemia is a known side-effect in high-dose Cisplatin chemotherapy of solid tumors [1,2]. In the classic case, hypocalcaemia is caused by excessive urinary loss and decreased renal up-take during high-dose Cisplatin treatment. Proximal tubular damage leads to decreased reabsorption of cations. Acute nephrotoxicity presents with increased creatinine and persistent protein and electrolyte losses. Chronic nephrotoxicity is characterized by a decrease of glomerular filtration rate and a slightly elevated but persistent magnesium, potassium and calcium excretion [3]. Hypomagnaesemic induced hypocalcaemia is caused by inhibition of parathyroid hormone secretion, impaired calcium release from the bones and low tissue responses to PTH due to low magnesium levels. Cisplatin associated hypocalcaemia and hypercalciuria can not be influenced by calcium supplementation. Correction of magnesium blood levels usually should improve the hypocalcaemia [4]. Hypocalcaemia may be associated with tetany, depression, carpopedal spasm, neuromuscular excitability, cardiac arrythmias with prolonged Q-T interval and sudden death, making it a true oncological emergency [5].

Outside cytotoxic therapy hypocalcaemia has been observed after thyroid and parathyroid surgery, chronic renal failure, acute rhabdomyolysis and pancreatitis [6-9]. Cisplatin is often used in combinations with other agents. Up to date, treatment of solid tumors increasingly consists of a combination of chemo- and immunotherapy. Combined use of Cisplatin, Interferon alpha and other chemo-therapeutic agents is applied in head and neck, renal, bladder, esophageal and pancreatic cancer [10-13]. However low-dose Cisplatin combined with biological Interferon-alpha therapy has previously not been associated with severe hypocalcaemia in patients with pancreatic cancer.

We report on several cases of severe hypocalcaemia after low-dose Cisplatin, 5-FU and Interferon alpha therapy (CapRI) and review the literature [14].

## Case presentation

Starting in August 2004 up to the present we have assigned 47 patients with pancreatic cancer within a prospective randomized study according to the CapRI schedule, including 23 patients treated with a multimodality concept consisting of low-dose Cisplatin (30 mg/m^2^), 5-FU, Interferon alpha and external beam radiation. Before the treatment all patients had undergone complete resection of a histologically proven adenocarcinoma of the pancreas. The median age was 60 years, 29 patients were male and 18 female. All patients had a Karnofsky score above 70%, had pre-treatment laboratory values of Hb >9.0 g/%, WBC >3000 cells/mm^3^, platelets >75.000 cells/mm^3^, creatinine <1.5 mg/dl and bilateral renal function determined by abdominal CT. None of them showed signs of acute infection or was diagnosed with a severe nonmalignant disease. Informed consent was obtained from all of the patients. 15 of 23 patients treated with radio-chemo-immunotherapy developed a moderate to severe hypocalcaemia defined as Ca^2+^-levels below 1.9 mmol/l, 8 patients were asymptomatic.

Here, we report as an example the case of a 63 year old man with a history of chronic pancreatitis presented with recurrent jaundice in September 2004. An ERCP revealed mechanical cholestasis and an endoprothesis was placed. MRI imaging demonstrated a big mass in the head of the pancreas highly suspicious for pancreatic carcinoma. A pylorus preserving Whipple operation was performed and pathology disclosed a poorly differentiated ductal adenocarcinoma. The tumor infiltrated the duodenum and portal vein and invaded lymphatic vessels. Staging imaging revealed no visible visceral or pulmonary metastases or lymph node involvement. In November 2004 adjuvant radio-chemo-immunotherapy according to the CapRI schedule was administered after informed consent was obtained. Treatment was initiated with radiation (50.4 Gy over 6 weeks), Cisplatin 30 mg/m^2 ^once a week, 5-Fluorouracil continuous infusion 200 mg/m^2^/day and Interferon alpha 3 MU subcutaneously 3 times weekly [14]. Four weeks after starting the treatment the calcium levels declined within 2 days from 2.21 mmol/l to 1.64 mmol/l (NR 2.1 – 2.65 mmol/l). Other electrolytes, creatinine, urea and albumin were in the normal range. Oral substitution with Calciumgluconate 1000 mg/day 3 times a day was started. The patient was asymptomatic but complained about diarrhoea 3 times a day. Despite medical recommendation patient refused daily consultation and i.v. drug administration or fluids. 6 days later he presented with severe dehydration, tetany, paraesthesia and carpopedal spasm. Trousseau's sign was positive and patient collapsed in the outpatient department. An ECG showed sinus-rhythm with prolonged Q-T interval (0.5 sec) and ST depression (>0.5 mm), blood pressure was 110/65 mmHg. Laboratory analysis revealed potassium of 2.4 mmol/l (NR 3.5 – 4.8 mmol/l), calcium of 1.28 mmol/l, creatinine of 1.16 mg/dl (NR <1.3 mg/dl), urea of 23 mg/dl (NR <45 mg/dl), albumin of 32.3 g/l (NR 30 – 50 g/l) and protein of 53.7 g/l (NR 60 – 80 g/l). Magnesium level was not measured at this time point. LDH was increased but the other cardiac enzymes and TNT were normal. Calciumgluconate 10%, calcitriol 0.5 μg and potassium 40 mmol were administered twice daily. Intensive i.v. fluids and monitoring were started and the patient was transferred to the intensive care unit. Chemotherapy and radiation were discontinued at that time. Symptoms improved slowly and there was no evidence of acute renal failure. 4 days later ECG, hydration and electrolytes were back to normal, but calcium was still low at 1.61 mmol/l. I.v. and oral substitution was continued and four weeks later the calcium level was 2.34 mmol/l. Chemotherapy without Cisplatin and Interferon was started again according to cycle 2 and 3 of the CapRI schedule, over the following weeks of treatment the calcium levels remained within the normal range and the patient was asymptomatic.

**Figure 1:**
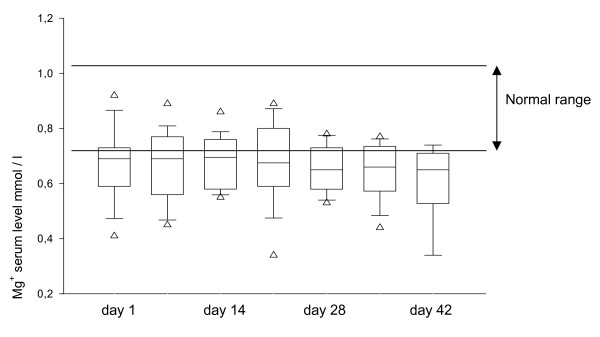
Boxplott of Magnesium serum levels. Day 1 after start of therapy to day 42.

**Figure 2:**
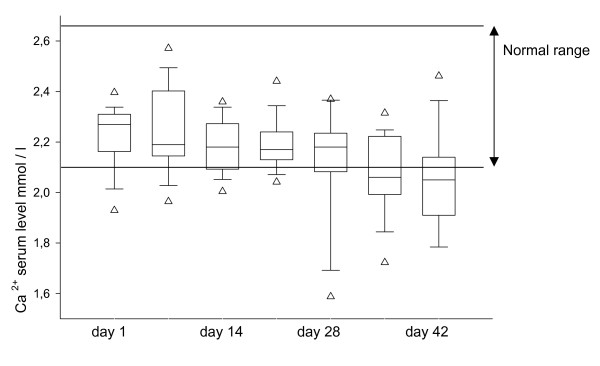
Boxplott of Calcium serum levels. Day 1 after start of therapy to day 42.

**Figure 3:**
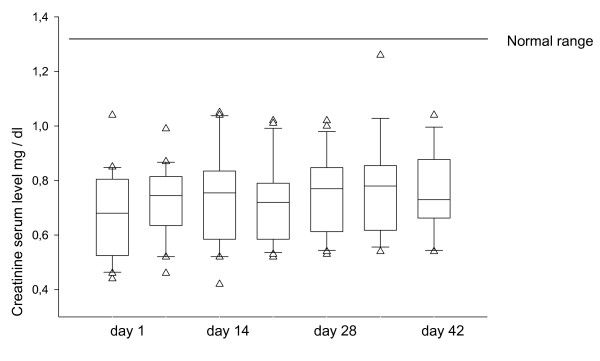
Boxplott of Creatinine serum levels. Day 1 after start of therapy to day 42.

The consecutive analysis of 23 patients treated with the CapRI schedule of combined radio-chemo-immunothrapy revealed mild to severe hypocalcaemia and hypomagnesaemia in 65% of the patients despite calcium and magnesium supplementation. Starting at day 14 of the therapy magnesium levels declined under normal range values (NR 0.75 – 1.05 mmol/l) with a median value of 0.65 mmol/l (SD ± 0.12 mmol/l). (Figure 1) The serum calcium levels of these patients reached values below the normal range at day 36 of treatment despite either oral or i.v. substitution started when the y were symptomatic with median levels of 2.04 mmol/l (SD ± 0.21 mmol/l). (Figure 2) During the whole course of radio-chemo-immunotherapy creatinine and albumin levels of the patients were within the normal range levels (median creatinine 0.66 ± 0.16 mg/dl, median albumin 40.8 ± 4.77 g/l). (Figure 3) Parathormone and 25-OH-Vitamin D3 levels were normal or slightly elevated. In addition to the reported patient, 6 patients with calcium levels <1.9 mmol/l complained of paraesthesia and mild tetany, 2 patients showed signs of depression, mental irritability and perioral numbness whereas 8 patients with calcium levels between 1.9 and 2.1 mmol/l were asymptomatic. (Table 1) 8 of the 15 patients with detected hypocalcaemia and hypomagnesaemia were insulin dependent diabetics. Compared to the non-diabetic group the calcium level of these patients was lower with median levels of 1.97 mmol/l (SD ± 0.21 mmol/l) between week three to six. Magnesium levels were decreased compared to the non-diabetic patients with median values of 0.58 mmol/l (SD ± 0.14 mmol/l). The albumin concentration (median value 38.6 ± 5.3 g/l) was comparable to the non-diabetic patients were as the creatinine level (median value 0.86 ± 0.2 mg/dl) was slightly increased. In all patients normal calcium levels were reached under i.v. and intensified oral therapy within 4 weeks of initial presentation.

## Discussion

Although oncologists commonly observe mild hypocalcaemia in high dose Cisplatin therapy, severe hypocalcaemia has not been associated with low dose Cisplatin treatment. Furthermore, review of the literature shows that hypocalcaemia has yet not been reported in patients with pancreatic carcinoma independent of the type of chemotherapy.

The patients reported on here received a novel, highly active treatment regimen by combining biologic and cytotoxic therapies. Picozzi et al. at Virginia Mason Medical Center demonstrated a prolongation of disease free and median survival with this approach [11,15].

Prior to the patient's treatment there have been no signs of electrolyte disturbance, up until the third week of treatment electrolytes were stable within the normal range. Despite kidney protection in the form of intensive hydration before and after Cisplatin administration severe decrease in calcium levels were observed within 48 hours after the fourth weekly dose without any sign of acute renal failure. At the time of therapy there was no evidence of tumor burden in these patients so hypocalcaemia due to tumor lysis syndrome could be excluded. The albumin was within the normal range in all patients during the whole therapy which excludes artificially low calcium concentrations due to a decrease of measurable protein-bound calcium fraction. However the incompliance of the reported patient might have aggravated the severity of the electrolyte disturbances. This implies that intensive medical advice is necessary to address the problem to the patients and the attending physicians.

Under normal conditions concentrations of calcium are highly modulated by the interplay between parat hormone (PTH), calcitonin and vitamin D. In hypocalcaemic conditions parat hormone and 25-OH-Vitamin D3 are upregulated. In our study population PTH and 25-OH-Vitamin D3 were slightly increased or normal during the hypocalceamic phase, a significant drop in PTH levels which might be causal for the hypocalceamia was not seen. Distal renal tubular damage by Cisplatin probably induces hypomagnesaemia due to DNA binding and genetic alteration of the magnesium transport [16,17]. Magnesium normally interacts with distal tubular calcium receptors increasing the reabsorption of both calcium and magnesium. Low serum concentrations of calcium will normally activate PTH excretion, but the increased gastrointestinal calcium up-take will be insufficiently in nauseated or vomiting patients. This and low tissue responses to PTH due to low magnesium levels might be responsible for severe hypocalcaemia in patients with pancreatic cancer treated with the CapRI scheme.

In 1979 Hayes et al. reported for the first time tetany in a series of 22 children with solid tumors under treatment with high dose Cisplatin (90 mg/m^2^) [18]. The possibility that the abnormalities reported were in part induced by the Interferon alpha has to be discussed. The mechanisms of nephrotoxicity induced by Interferon alpha are unclear. However, mild proteinuria (25%), elevated serum creatinine (10%) and blood urea nitrogen (10%) were reported by Jones et al. in 1300 patients treated with Interferon alpha [19]. Intensive electrolyte wasting syndromes were reported in patients with head and neck cancer treated with Cisplatin (100 mg/m^2^), continuous 5-FU infusion and escalating doses of Interferon alpha. Despite intensified supportive care and iv replacement 4 patients died during phase one of the study from dehydration, diarrhoea and loss of electrolytes. Although these are known side effects of high dose Cisplatin therapy Vokes et al. concluded that Interferon and Cisplatin might have synergistic toxic tubular effects [13]. At Virginia Mason, Picozzi et al. did not report any hypocalcaemia using the same treatment schedule as our group [13].

However, the sudden decrease in calcium levels in our patients suggests that low dose Cisplatin toxicity alone would be an unlikely explanation for the observed events. Regarding the experience in other combined adjuvant radio-chemotherapy for pancreatic cancer it seems that radiation has no synergistic effect on Cisplatin-induced hypocalceamia [20,21]. It is noteworthy that diabetic patients were identified to be at significantly increased risk of developing high levels of serum creatinine and hypocalcaemia [13]. Patients after pancreatic surgery often present with postoperative diabetes and represent a high risk group for side effects during Cisplatin and Interferon based chemotherapy. Diabetic patients presented in our study with lower calcium and magnesium and increased creatinine levels, what can confirm this hypothesis. A decreased clearance of Cisplatin and Interferon due to diabetic renal malfunction in these patients has to be discussed as a reason.

## Conclusion

Using this therapy approach the authors strongly recommend intensive electrolytes monitoring and prophylactic treatment of every patient with continuous oral calcium and magnesium substitution starting at normal electrolyte levels. In nauseated patients and patients with severe dehydration, diarrhea or vomiting early i.v. fluid and electrolyte administration have to be considered. In patients with symptomatic hypocalcaemia and hypomagnesaemia i.v. substitution is mandatory.

## Competing interests

The author(s) declare that they have no competing interests.

## Authors' contributions

KH acquired and analyzed the data. Medical care is covered by KH, KL, SF. AM, JS, MWB planned, coordinated and conducted the study. DJ revised the manuscript. Scientific program is planned by AM and carried out by AM and KH. All authors read and approved the final manuscript.

## Pre-publication history

The pre-publication history for this paper can be accessed here:



## Figures and Tables

**Table 1:** Symptoms and electrolyte values of symptomatic patients. Normal ranges: Calcium 2,1 – 2,65 mmol/l, Magnesium 0,75 – 1,05 mmol/l, Albumin 30 – 50 g/l, Creatinine <1,3 mg/dl, *: out of range

***Patient***	***Symptoms***	***Calcium***	***Magnesium***	***Albumin***	***Creatinine***
		***(mmol/l)***	***(mmol/l)***	***(g/l)***	***(mg/dl)***

1	tetany, carpopedal spasm, paraesthesia	1,28*	~	32,3	1,16
2	paraesthesia	1,84*	~	37,5	0,93
3	paraesthesia, tetany	1,84*	0,71*	30,1	0,82
4	paraesthesia, depression	1,8*	0,77	39,4	0,62
5	paraesthesia	1,62*	0,56*	32,3	0,71
6	paraesthesia, tetany	1,69*	0,29*	37,2	1,04
7	paraesthesia, depression	1,88*	0,42*	27,5*	0,7
